# Comparison of standard staging protocol and WB-MRI for initial staging of rectal cancer

**DOI:** 10.1186/1470-7330-15-S1-S9

**Published:** 2015-10-02

**Authors:** JH Yoon, JM Lee, MH Yu, BY Hur, JK Han

**Affiliations:** 1 Seoul National University Hospital, Seoul, South Korea; 2 Kon-kuk University Hospital, Seoul, South Korea; 3 National Cancer Center, Goyang, South Korea

## Aim

To evaluate the clinical feasibility of whole-body magnetic resonance imaging (WB-MRI) including contrast-enhanced T1-weighted imaging (T1WI) and diffusion-weighted whole-body imaging with background body signal suppression (DWIBS) in rectal cancer initial staging

## Methods

This retrospective study was approved by our IRB and the requirement for informed consent was waived. A total of 133 patients (M:F=87:46, mean age 62.4 years) who underwent standard protocol (chest, abdomen computed tomography and rectal MRI), WB-MRI (WB-T1WI and DWIBS) at 3T for initial staging were included. One attending radiologist reviewed standard protocol and two attending radiologists reviewed WB-MRI in consensus. Finally, “true” M staging was obtained using either biopsy or follow-up imaging. Agreement of M-staging for rectal cancer was obtained between standard protocol and WB-MRI, between true M-stage and standard-protocol and WB-MRI.

## Results

The agreement for M-staging between standard protocol and WB-MRI was 83.5% (111/133). M- staging of WB-MRI agreed to that of standard protocol in 96.0% (97/101) for M0, and 43.7% (14/32) for M1. M-staging agreement between standard protocol and “true” M-staging was 86.5% (115/133): standard protocol agreed to “true” M-staging in 86.8% (99/114) for M0 and in 88.9% (16/18) for M1. WB-MRI showed 94.0% (125/133) of agreement to “true” M-staging: the agreement rates between the two were 97.4% (111/114) for M0 and 77.8% (14/18) for M1. One patient who was reported as having lung metastasis on both protocols was confirmed with primary lung cancer on biopsy.

## Conclusion

WB-MRI showed high agreement with standard protocol for initial rectal cancer staging and “true” M-staging.

